# Adeno-associated virus expressing a blood-brain barrier–penetrating enzyme improves GM1 gangliosidosis in a preclinical model

**DOI:** 10.1172/JCI180724

**Published:** 2025-04-08

**Authors:** Saki Kondo Matsushima, Yohta Shimada, Masafumi Kinoshita, Takashi Nagashima, Shinichiro Okamoto, Sayoko Iizuka, Haruna Takagi, Shunsuke Iizuka, Takashi Higuchi, Hiroyuki Hioki, Ayako M. Watabe, Hiroyuki Sonoda, Toya Ohashi, Hiroshi Kobayashi

**Affiliations:** 1 Division of Gene Therapy, Research Center for Medical Sciences, The Jikei University School of Medicine, Minato-ku, Tokyo, Japan.; 2 Research Division, JCR Pharmaceuticals, Kobe, Hyogo, Japan.; 3 Institute of Clinical Medicine and Research, Research Center for Medical Sciences, The Jikei University School of Medicine, Kashiwa, Chiba, Japan.; 4 Department of Neuroanatomy, Juntendo University Graduate School of Medicine, Bunkyo-ku, Tokyo, Japan.; 5 Advanced Research Institute for Health Science, Juntendo University, Bunkyo-ku, Tokyo, Japan.; 6 Department of Cell Biology and Neuroscience and; 7 Department of Multi-Scale Brain Structure Imaging, Juntendo University Graduate School of Medicine, Bunkyo-ku, Tokyo, Japan.; 8 Department of Human Health Science and Therapeutics, The Jikei University of School of Nursing, Komae, Tokyo, Japan.

**Keywords:** Genetics, Therapeutics, Gene therapy, Lysosomes

## Abstract

GM1 gangliosidosis is a lysosomal storage disorder (LSD) caused by genetic defects in lysosomal β-galactosidase (β-gal). The primary substrate of β-gal is GM1 ganglioside (GM1), a sialylated glycosphingolipid abundant in the central nervous system (CNS). Deficiency in β-gal causes GM1 to accumulate in neural cells, leading to a rapid decline in psychomotor functions, seizures, and premature death. There is currently no therapy available. Although enzyme replacement therapy has been approved for other LSDs, its effects on the CNS are limited owing to the blood-brain barrier (BBB). Here, we assessed the therapeutic efficacy of a systemic infusion of an adeno-associated virus vector carrying a gene expressing a BBB-penetrable enzyme under the control of a liver-specific promoter in GM1 gangliosidosis model mice. The BBB-penetrable enzyme consisted of the variable region of the anti–transferrin receptor antibody fused with β-gal. The BBB-penetrable enzyme was only produced in the liver and secreted into the blood, which was efficiently distributed to various organs, including the brain. GM1 accumulation in the CNS was completely normalized, with improved neurological functions and animal survival. This therapeutic approach is expected to be applied for the treatment of several hereditary neurological diseases with CNS involvement.

## Abstract 2

<strong>Complete normalisation of GM1 ganglioside storage through systemic infusion of AAV vector expressing a blood-brain barrier-penetrating enzyme in a murine model of GM1 gangliosidosis</strong>

## Graphical abstract

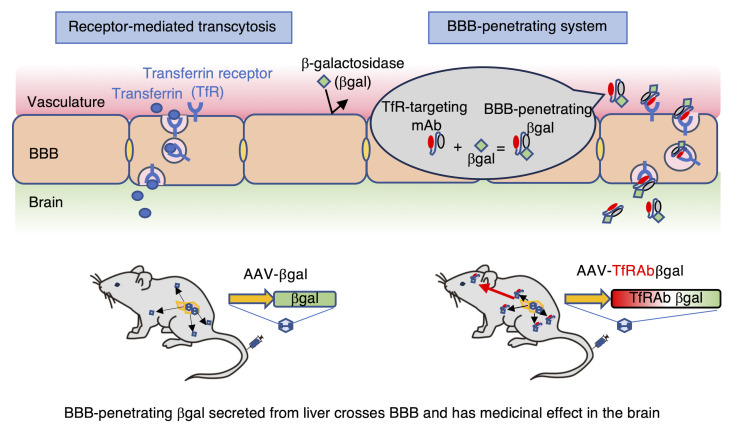

## Introduction

Lysosomal storage disorders (LSDs) are one of the most common monogenic neurodegenerative diseases in children, with an incidence of approximately 1 in every 8,000 births worldwide (1). Most LSDs are caused by a defect in a single enzyme gene that results in the accumulation of the undegraded substrates. This substrate accumulation results in a broad spectrum of clinical manifestations.

GM1 gangliosidosis (OMIM 230500) is an LSD caused by genetic defects in the *GLB1* gene, which encodes a lysosomal enzyme, β-galactosidase (β-gal), that hydrolyzes the nonreducing terminal β-galactosyl residues of GM1 ganglioside (GM1), glycoproteins, and glycosaminoglycans (2). Deficiency in β-gal leads to the accumulation of GM1 and its asialo derivative GA1 mostly in the central nervous system (CNS) (3). In the peripheral organs, partially degraded keratan sulfate, oligosaccharides, and glycoproteins accumulated (4), causing skeletal dysplasia, hepatosplenomegaly, and cardiomyopathy. The incidence of GM1 gangliosidosis is estimated to be 1 in 100,000 to 200,000 live births (5), and GM1 gangliosidosis is classified into 3 subtypes — infantile, juvenile, and adult — according to the onset and severity of symptoms (6, 7). The infantile form (type I), the most severe subtype, causes developmental delay by 3–6 months of age and presents with hypotonia, hypersensitivity to sound, generalized convulsions, cherry red spots on the fundus, hepatosplenomegaly, and bone abnormalities throughout the body. Usually the patient dies by the age of 2. In the late-infantile or juvenile form (type II), clinical symptoms are similar to those of the infantile form but somewhat milder. Patients with the adult form (type III) have normal development or minimal intellectual disability. Abnormal gait and dysarthria are the most common initial symptoms of GM1 gangliosidosis.

The newly synthesized lysosomal enzyme is mainly localized in the lysosome, but part of the lysosomal enzyme is excreted into the outside of cells. In addition, the extracellular lysosomal enzyme is taken up through receptor-mediated endocytosis into the cells and localized in the lysosome. This unique feature of the lysosomal enzyme, called cross-collection mechanism, enables enzyme replacement therapy (ERT) (8). In ERT, a commonly used therapy for LSDs, the defective enzyme is administered intravenously as a recombinant protein. It has been approved for Gaucher disease, mucopolysaccharidosis (MPS) I, II, IV, VI, and VII, Fabry disease, neuronal ceroid lipofuscinosis, acid sphingomyelinase deficiency, and Pompe disease. However, the native enzyme does not penetrate the blood-brain barrier (BBB), making ERT ineffective for the treatment of CNS symptoms of LSDs. In this regard, the direct infusion of the enzyme into the brain and the intravenous infusion of the BBB-penetrating enzyme are approved for MPS II (9–14) and neuronal ceroid lipofuscinosis (15–17). Another limitation of ERT is the requirement of repeated infusions throughout life. Other therapeutic approaches for LSDs are pharmacological chaperones and substrate reduction therapy (SRT). The great advantage of these small-molecule therapies is that they are orally administered drugs. Pharmacological chaperones bind and stabilize misfolded proteins and contribute to increasing the residual activity. This approach is approved only for Fabry disease (18–20) and has been extensively investigated for Gaucher disease (21, 22) and GM1 gangliosidosis (23–25). The main limitation of pharmacological chaperone therapy is that it is only effective in patients with amenable mutations. SRT consists of inhibiting the synthetic enzyme, resulting in a reduction of the accumulated substrates, and has been approved for Gaucher disease and Niemann-Pick type C disease. A phase IV clinical trial is ongoing for GM1 and GM2 gangliosidosis (ClinicalTrials.gov NCT02030015). One of the 2 approved drugs for Gaucher disease is available only after genotyping has been conducted to determine the cytochrome P450 mutation (26–28).

To overcome the limitations of current treatments for LSDs, gene therapy has been investigated, and several gene therapy products for inherited metabolic diseases have already been approved, including therapies for metachromatic leukodystrophy (29, 30) and adrenoleukodystrophy (31, 32). Gene therapy has an advantage over current LSD therapy because it does not require repeated administration and can be applied to all patients regardless of gene mutations. In gene therapy, the cells transduced by the vector carrying the normal lysosomal enzyme gene excrete the lysosomal enzyme extracellular space, which is taken up by neighboring cells via a mannose-6-phosphate receptor on the cell surface. Thus, not all cells need to be transduced by the gene. This cross-correction mechanism is an advantage of the gene therapy approach to treat LSDs.

As with other LSDs, several preclinical gene therapy studies have been reported using GM1 gangliosidosis animal models, including the in vivo infusion of adenovirus (33) and adeno-associated virus (AAV) vectors (34–38) and the ex vivo modification of hematopoietic stem cells with retrovirus (39) and lentivirus vectors (40). Furthermore, 2 phase I/II clinical trials on safety and efficacy in GM1 gangliosidosis are ongoing, one to study a single-dose intravenous administration of AAV9/*GLB1* (ClinicalTrials.gov NCT03952637) and the other to study a single-dose intra–cisterna magna administration of AAVhu68/*GLB1* (ClinicalTrials.gov NCT04713475). The major clinical burden of patients with GM1 gangliosidosis is the CNS symptoms, highlighting the need to investigate effective, noninvasive alternative gene therapies.

The BBB consists of a continuous monolayer of brain capillary endothelial cells, pericytes, and astrocytes (41). The BBB is both a physical barrier and a selective transport system that regulates the passage of molecules that are necessary for the brain. Several endogenous molecules, such as insulin, leptin, and transferrin, can pass through the BBB by transcytosis via their specific receptors expressed on the luminal side of brain capillary endothelial cells (42–44). Several preclinical trials using this BBB-penetrating system were conducted to allow lysosomal enzymes to reach the brain and treat brain lesions in lysosomal disease (10, 45–48). Recently, a lysosomal enzyme product fused with anti–human transferrin receptor antibody (anti-hTfRAb) has been approved as ERT for MPS II in Japan (9).

Despite numerous efforts, no effective treatment has been developed for GM1 gangliosidosis. The combination of the BBB-penetrating system and AAV vector will have a significant advantage because repeated administrations will not be necessary and the continuous secretion of the enzyme might be more effective than the intermittent administration of the enzyme. Since transferrin receptor (TfR) is expressed throughout the body, the uptake of the fusion protein is increased in various organs as well as the brain. This system has the potential to overcome all the issues of conventional treatment methods.

Here, we report the treatment of CNS symptoms in GM1 gangliosidosis model mice using intravenously administered AAV vector expressing a fusion protein, Tβ-gal, consisting of a rat monoclonal antibody against mouse transferrin receptor (anti-mTfRAb) and human β-gal.

## Results

### Overview of the study.

The therapeutic efficacy of systemic AAV-β-gal (G) or AAV-Tβ-gal (T) delivery was evaluated in adult GM1 mice infused with 1 × 10^^12^^ (low dose; Low) or 5 × 10^^12^^ (high dose; High) viral genomes (vg)/kg via the tail vein at 10 weeks of age. Each transgene is expressed under the control of a liver-specific promoter. Controls included untreated GM1 mice (NT), age-matched normal wild-type mice (WT), and heterozygote mice (HZ). The enzymatic activity in the serum was measured over time after the administration. Behavioral testing was performed at 32 or 33 weeks of age. A subset of mice from the AAV-treated and control groups were euthanized at 33 weeks of age for biochemical and histological studies.

### Tβ-gal is overexpressed in the liver and penetrates all over the body, including the CNS.

**Figure 1:**
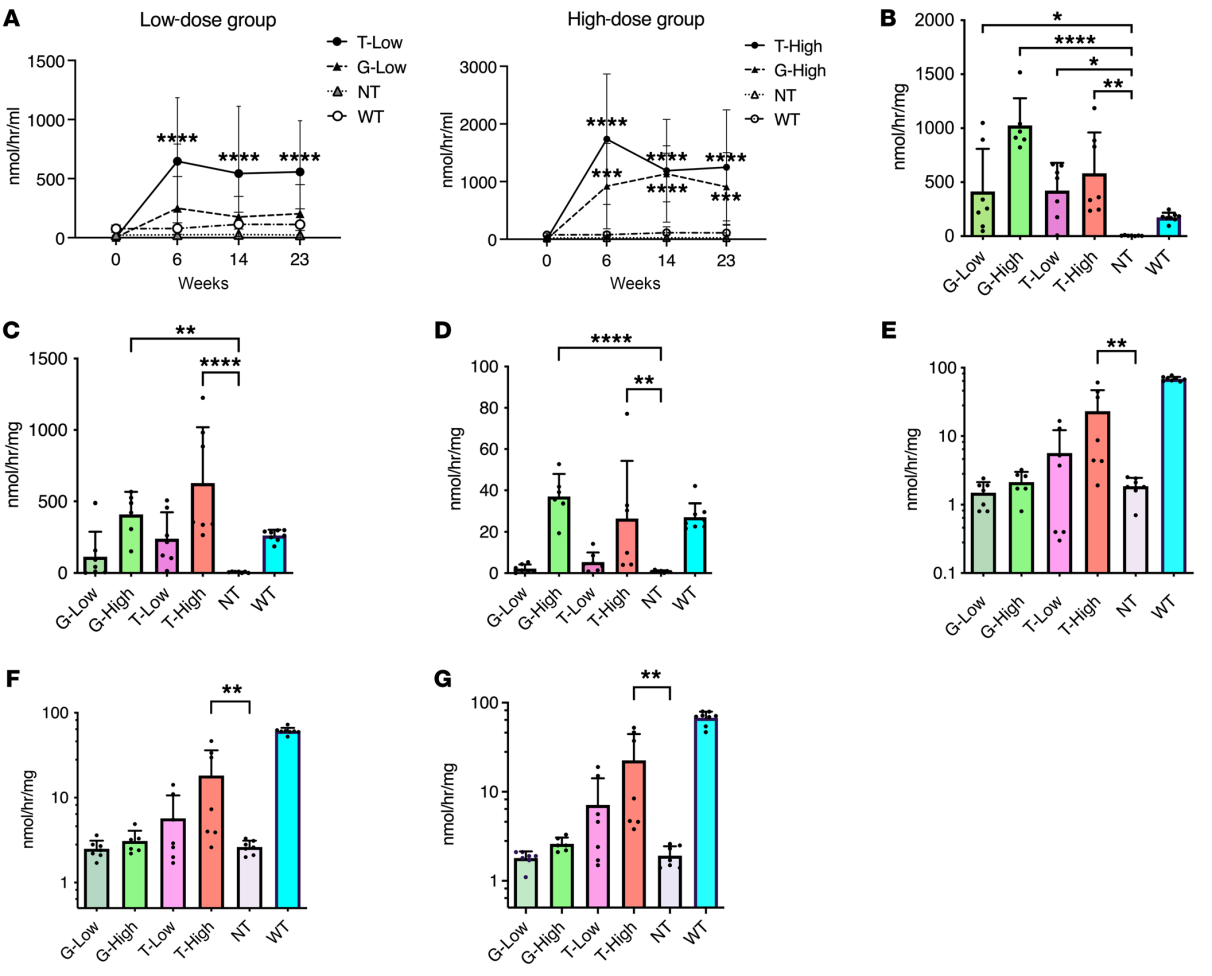
β-Gal activity in systemic organs. β-Gal activity in various tissues was analyzed at 23 weeks after treatment: (**A**) serum (*n* = 10 for NT group, *n* = 12 for G-High group, *n* = 13 for G-Low, T-Low, and T-High groups, *n* = 17 for WT group), (**B**) liver, (**C**) spleen, (**D**) heart, (**E**) cerebrum, (**F**) cerebellum, and (**G**) hippocampus (*n* = 6 for G-High group, *n* = 7 for G-Low, T-Low, T-High, and NT groups, *n* = 8 for WT group). The β-gal activity was expressed as nanomoles per hour per milliliter of serum and as nanomoles per hour per milligram of protein in tissues. One mouse in the T-Low group did not show any increase in β-gal activity in all organs we measured after treatment, resulting in a large error bar. G-Low, 1 × 10^12^ vg/kg of AAV-β-gal treatment; G-High, 5 × 10^12^ vg/kg of AAV-β-gal treatment; T-Low, 1 × 10^12^ vg/kg of AAV-Tβ-gal treatment; T-High, 5 × 10^12^ vg/kg of AAV-Tβ-gal treatment; NT, nontreated GM1 mice; WT, wild-type mice. Results are shown as means ± SD. Significance was evaluated by a 1-way or 2-way ANOVA followed by Dunnett’s multiple-comparison test. *P < 0.05, **P < 0.005, ****P < 0.0001 vs. NT.

The enzyme released from the transgenic hepatocyte cells into the blood maintained high concentrations throughout the experiment, i.e., until 23 weeks after administration (Figure 1A and Supplemental Figure 1A; supplemental material available online with this article; https://doi.org/10.1172/JCI180724DS1). The enzymatic activity in the serum was markedly higher in a dose-dependent manner and reached 10- to 27-fold higher levels in the low-dose groups (G-Low, T-Low) and 38 to 52 times higher levels in the high-dose groups (G-High, T-High) as compared with the NT group. The enzymatic activity in the T-Low group was higher than that in the G-Low group, though the specific activity of Tβ-gal was much lower than that of β-gal (Supplemental Figure 1B). In addition, the β-gal protein concentration in the serum was higher in the T groups than in the G groups (Supplemental Figure 1C). In summary, the transgenic enzyme secretion levels in the T groups were higher than those in the G groups.

Among the peripheral organs, the enzymatic activity was not substantially different if the same dose of G and T was administered (Figure 1, B–D). In the CNS, the BBB prevents β-gal from penetrating the brain. Thus, there was not markedly increased enzymatic activity in both G-Low and G-High groups compared with the NT group in the CNS (Figure 1, E–G). In contrast, the T-Low and T-High groups showed higher levels of enzymatic activity in the CNS than the NT group, especially the T-High group, whose enzymatic activity was increased by 43%, 37%, and 41% compared with that of the WT group in the cerebrum, cerebellum, and hippocampus, respectively. These results strongly suggested that Tβ-gal was expressed in the liver and eventually penetrated the brain via TfR-mediated transcytosis.

### Normalization of accumulated GM1 contents in the brain mediated by T.

**Figure 2:**
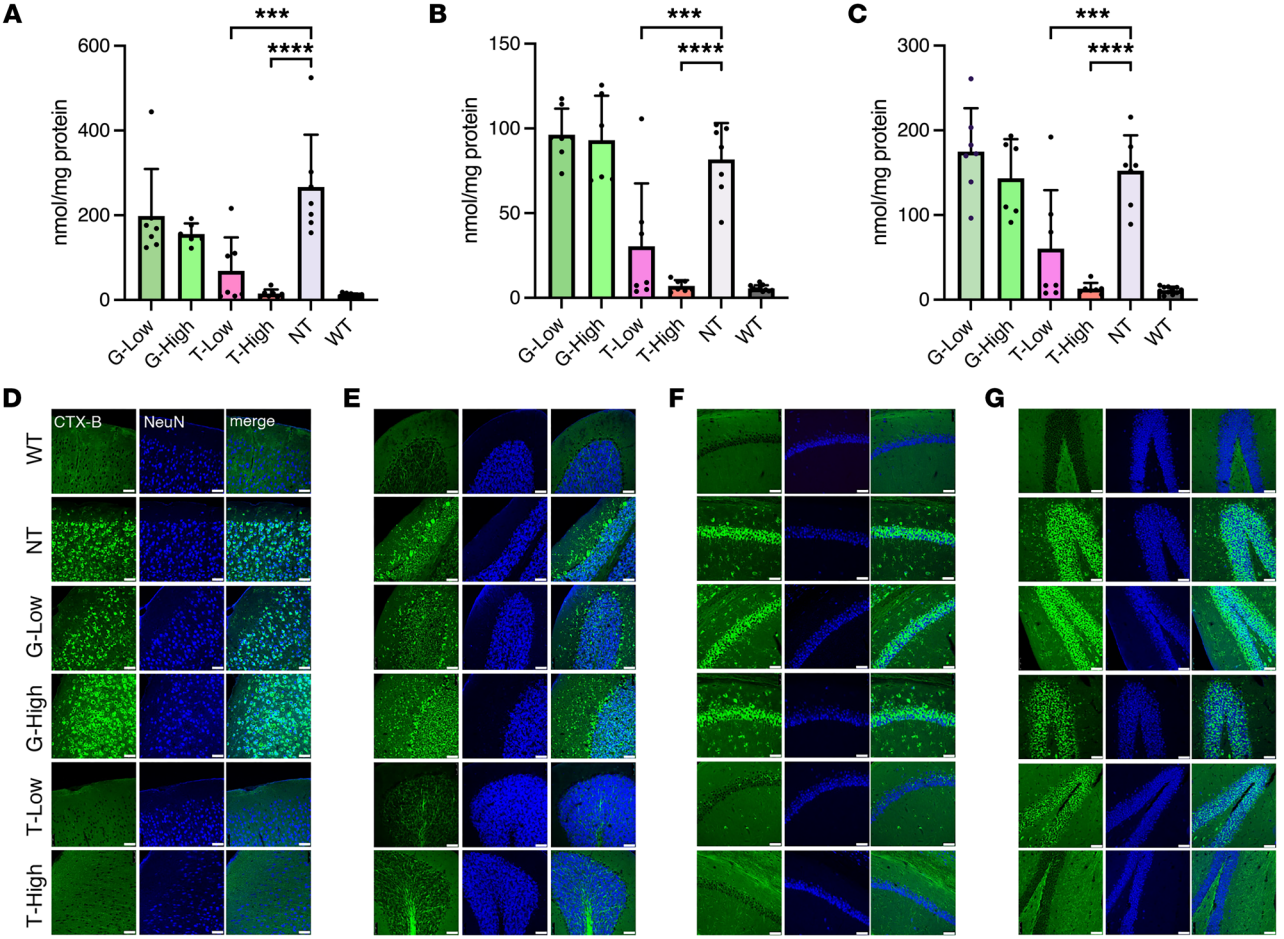
GM1 content storage in the CNS. GM1 storage of the cerebrum, cerebellum, and hippocampus was evaluated by LC-MS/MS and immunostaining. (**A**–**C**) GM1 content in the cerebrum (**A**), cerebellum (**B**), and hippocampus (**C**) measured by LC-MS/MS (*n* = 6 for G-High group, *n* = 7 for G-Low, T-Low, T-High, and NT groups, *n* = 11 for WT group). One mouse in the T-Low group did not show any increase in β-gal activity in all organs we measured after treatment, resulting in a large error bar. (**D**–**G**) Immunostaining of selected brain regions: M1 region of cerebrum (**D**), cerebellum (**E**), and cornu ammonis 1 (CA1) (**F**) and dentate gyrus (DG) (**G**) regions of hippocampus. Green, CTB-X staining; blue, NeuN staining. Representative images are shown following staining of 3 per group. Scale bars: 50 μm. G-Low, 1 × 10^12^ vg/kg of AAV-β-gal treatment; G-High, 5 × 10^12^ vg/kg of AAV-β-gal treatment; T-Low, 1 × 10^12^ vg/kg of AAV-Tβ-gal treatment; T-High, 5 × 10^12^ vg/kg of AAV-Tβ-gal treatment; NT, nontreated GM1 mice; WT, wild-type mice. Results are shown as means ± SD. Significance was evaluated by a 1-way ANOVA followed by Dunnett’s multiple-comparison test. ***P < 0.001, ****P < 0.0001 vs. NT.

To analyze the amounts and distribution of GM1 in different brain regions, we used liquid chromatography–tandem mass spectrometry (LC-MS/MS) and immunofluorescence staining. In the T-High group, a significant reduction of GM1 isoform (C18) was observed in the cerebrum, cerebellum, and hippocampus (*P* = 0.0014, 0.0001, and 0.0008, respectively, as compared with that in the NT group; Figure 2, A–C). The level was almost equal to that observed in the WT mice. In the T-Low group, a significant reduction was also observed in the cerebrum and cerebellum (*P* = 0.0127 and 0.0409, respectively). One mouse in the T-Low group showed no increase in β-gal activity after infusion, and thus its GM1 accumulation was at the same level as that observed in the NT group. The G-High group showed a marginal reduction in the cerebrum and hippocampus (55% and 82% of the levels in the NT group, respectively); however, this was not substantially different from the NT group. These results are consistent with the results of the β-gal activity shown in Figure 1.

To histologically examine the distribution of GM1 storage in the brain, we stained brain sections (cerebral cortex [M1], cerebellum, and hippocampus [cornu ammonis 1 and dentate gyrus]) with cholera toxin B (CTX-B) for GM1 staining and with anti-NeuN antibody, a neuron marker (Figure 2, D–G). The CTX-B^^+^^ neuron (NeuN^^+^^ cell) was not observed in the brain sections of the WT mice, and strong CTX-B^^+^^ neuron signals were observed in those of the NT mice. In the G groups, strong CTX-B^^+^^ neuron signals were observed in all the tested brain areas. The CTX-B^^+^^ neuron was not observed in the T-High group. In the T-Low group, the CTX-B^^+^^ neuron was not observed in the cortex or cerebellum, but was observed in the hippocampus, though the signal was lower than that in the NT group.

The combined results of the LC-MS/MS and CTX-B staining strongly indicated that gene therapy using T, especially using high-dose T, completely normalized the accumulation of GM1 in the CNS.

### Reduction in inflammation in the brain of T-treated mice.

**Figure 3:**
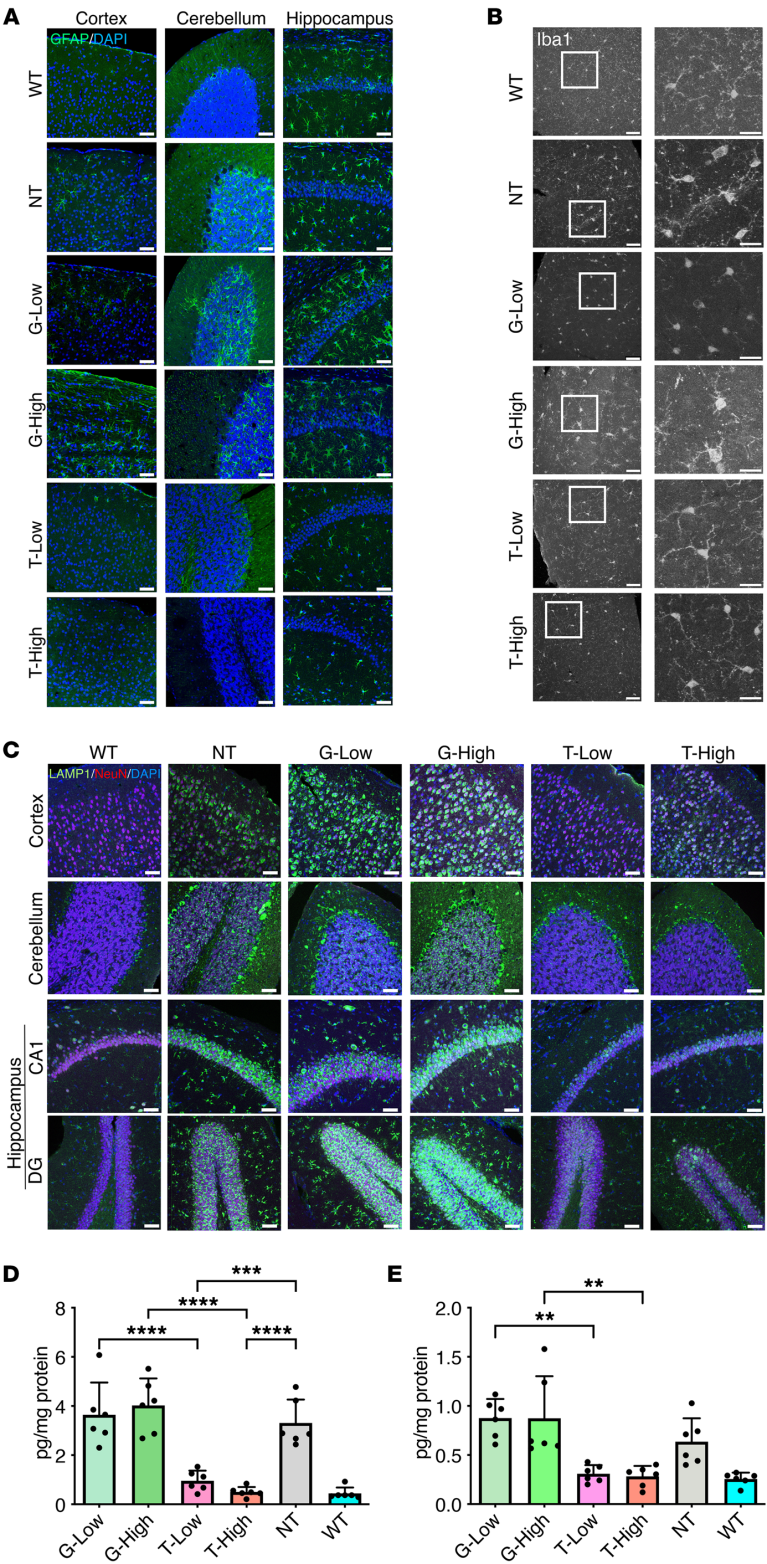
Neuropathological features. (**A**) Astrocytosis was evaluated with immunostaining using anti-GFAP antibody. GFAP (green) and DAPI (blue) staining in the cerebrum, cerebellum, and hippocampus. (**B**) Staining with Iba1 (gray). Images on the right show ×3 zoom of white boxes in overview images. (**C**) Staining with LAMP1 (green), NeuN (red), and DAPI (blue). Representative images are shown following staining of 3 per group. (**D** and **E**) Quantification of cytokines MIP-1a and IP-10 in the brain at 33 weeks of age (*n* = 6 per groups). G-Low, 1 × 10^12^ vg/kg of AAV-β-gal treatment; G-High, 5 × 10^12^ vg/kg of AAV-β-gal treatment; T-Low, 1 × 10^12^ vg/kg of AAV-Tβ-gal treatment; T-High, 5 × 10^12^ vg/kg of AAV-Tβ-gal treatment; NT, nontreated GM1 mice; WT, wild-type mice. Scale bars: 50 μm and 25 μm (×3 zoom). Results are shown as means ± SD. Significance was evaluated by a 1-way ANOVA followed by Tukey’s multiple-comparison test. **P < 0.005, ***P < 0.001, ****P < 0.0001.

To examine whether the normalization of GM1 accumulation would reduce a neuroinflammatory response characterized by prominent astrogliosis and microgliosis in GM1 mice, brain sections were stained with an antibody against glial fibrillary acidic protein (GFAP), a marker of astrocytes, and an antibody against ionized Ca^^2+^^-binding adaptor molecule 1 (Iba1), a marker of microglia. Faint GFAP^^+^^ cells were observed in WT mice, whereas a number of GFAP^^+^^ reactive astrocytes were stained in the cerebrum and cerebellum of NT mice. Similar results were observed in the G groups; however, a clear decrease in GFAP^^+^^ cells was observed in the T groups. Especially in the T-High group, GFAP^^+^^ cells were reduced to the same level as in the WT group (Figure 3A). The shape of activated microglia is ameboid with small branches, whereas the shape of inactive microglia is dendritic. In the WT mouse brains, the Iba1^^+^^ microglia showed an inactive form, forming dendrites, and in the T groups, most of the microglia were of the inactive form, i.e., the ramified microglia had extensive branching (Figure 3B). In the NT and G groups, most of the microglia were in the activated form.

The brain sections were also stained with lysosomal-associated membrane protein 1 (LAMP1) to highlight lysosomal swelling (Figure 3C). In the NT group, the NeuN^^+^^ cells exhibited markedly increased LAMP1 staining compared with those in the WT group, with intense large vesicular LAMP1^^+^^ lysosomes present in distinct locations around the nucleus in the CNS. In the T groups, dramatic reductions in LAMP1 signals were observed. In contrast, the LAMP1 expression in the G groups was not reduced and was very similar to that in the NT group.

A substantial upregulation of the MIP-1a and IP-10 proteins, which are known as inflammatory cytokines, was observed in the brains of the NT group as compared with the WT group at 33 weeks of age (Figure 3, D and E). The G treatment resulted in no change in the levels of these proteins, but the T treatment showed complete normalization of MIP-1a and IP-10 levels in the brain. Taken together, these results indicated that the T treatment lowered the neuroinflammatory response in the brain as compared with that in the NT group.

### Effect of T treatment on motor performance of GM1 mice.

**Figure 4:**
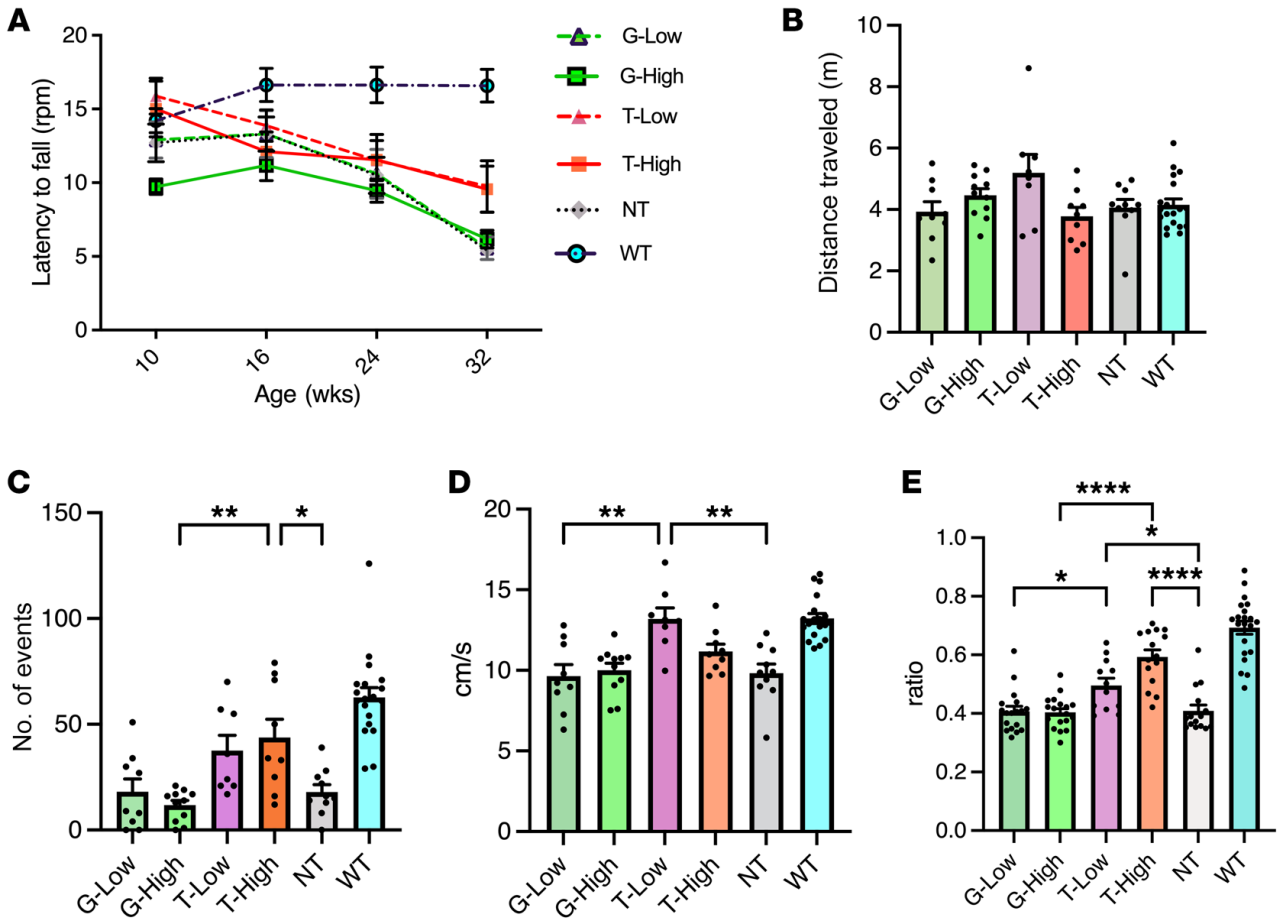
T improves several motor performances. Behavioral evaluation with rotarod test, open-field test, and gait analysis was conducted. (**A**) Rotarod testing: highest latency achieved on 4–40 rpm accelerating rotarod over 300 seconds over 3 trials (*n* = 8 for T-Low group, *n* = 10 for G-Low, T-High, and NT groups, *n* = 11 for G-High group, *n* = 19 for WT group). (**B**–**D**) Open-field test. Total distance traveled (**B**), rearing number (**C**), and moving speed (**D**) were analyzed (*n* = 8 for T-Low group, *n* = 9 for G-Low and T-High groups, *n* = 11 for G-High group, *n* = 10 for NT group, *n* = 19 for WT group). (**E**) Gait analysis (*n* = 12 for T-Low group, *n* = 15 for T-High and NT groups, *n* = 18 for G groups, *n* = 21 for WT group). Footprints were evaluated for stride length/body length ratio. G-Low, 1 × 10^12^ vg/kg of AAV-β-gal treatment; G-High, 5 × 10^12^ vg/kg of AAV-β-gal treatment; T-Low, 1 × 10^12^ vg/kg of AAV-Tβ-gal treatment; T-High, 5 × 10^12^ vg/kg of AAV-Tβ-gal treatment; NT, nontreated GM1 mice; WT, wild-type mice. Results are shown as means ± SEM. Significance was evaluated by a 1-way ANOVA followed by Tukey’s or Dunnett’s multiple-comparison test. *P < 0.05, **P < 0.005, ****P < 0.0001.

To evaluate the effects of the normalization of GM1 content and reduction of neurological inflammation in the brain on the locomotor performance of GM1 mice, we performed the rotarod test, open-field test, and gait analysis (Figure 4). In the rotarod test, the NT mice exhibited a decrease in motor function as compared with the WT mice at 32 weeks (Figure 4A). The mice in the T groups tended to exhibit a longer latency to fall from the rotarod compared with the NT group at 32 weeks, but the mice in the G groups did not. In the open-field test at 33 weeks, there were no significant differences in all the groups in terms of the total distance and time spent in center (Figure 4B and Supplemental Figure 2). The number of rearing events (Figure 4C) and moving speed (Figure 4D) of the NT mice were significantly decreased compared with those of the WT mice (*P* < 0.0001). The G groups showed results similar to those of the NT groups. On the other hand, T treatment significantly increased the rearing number (T-High, *P* = 0.0080) and moving speed (T-Low, *P* = 0.003) compared with those of the NT group. These results suggested that the T treatment had a partial therapeutic effect on motor function in GM1 mice, particularly on hind-limb muscle strength, which is important for standing movement. The mouse model of GM1 recapitulates the early-onset forms of the disease, with gradually deteriorating motor function eventually leading to hind-limb paralysis (49). In addition, GM1 accumulation is observed in the peripheral nerve of GM1 model mice (50) as well as in that of patients with GM1 gangliosidosis (51). To further investigate this result, we performed gait assays at 33 weeks of age (Figure 4E). The stride length/body length ratios of the NT and G groups were significantly lower than those of the WT (*P* < 0.0001) and T (T-Low, *P* = 0.036; T-High, *P* < 0.0001) groups, suggesting that the T treatment completely rescued the disease phenotype of reduced stride length in GM1 mice.

### Effects of T treatment on hematopoiesis.

Although the anti-mTfRAb binding site in TfR is different from the site to which endogenous transferrin originally binds, we assessed whether anti-mTfRAb inhibits transferrin binding to the receptor. The hemoglobin (HGB) level was lower in a dose-dependent manner in the T groups (Supplemental Figure 3A). The mean corpuscular volume (MCV) and mean corpuscular hemoglobin concentration (MCHC) were low in the T-High group (Supplemental Figure 3, B and C), and splenomegaly was also observed (Supplemental Figure 3D). These results suggested that the increased levels of anti-mTfRAb in the blood may inhibit endogenous transferrin incorporation into the cells.

### T treatment extends the lifespan of GM1 mice.

**Figure 5:**
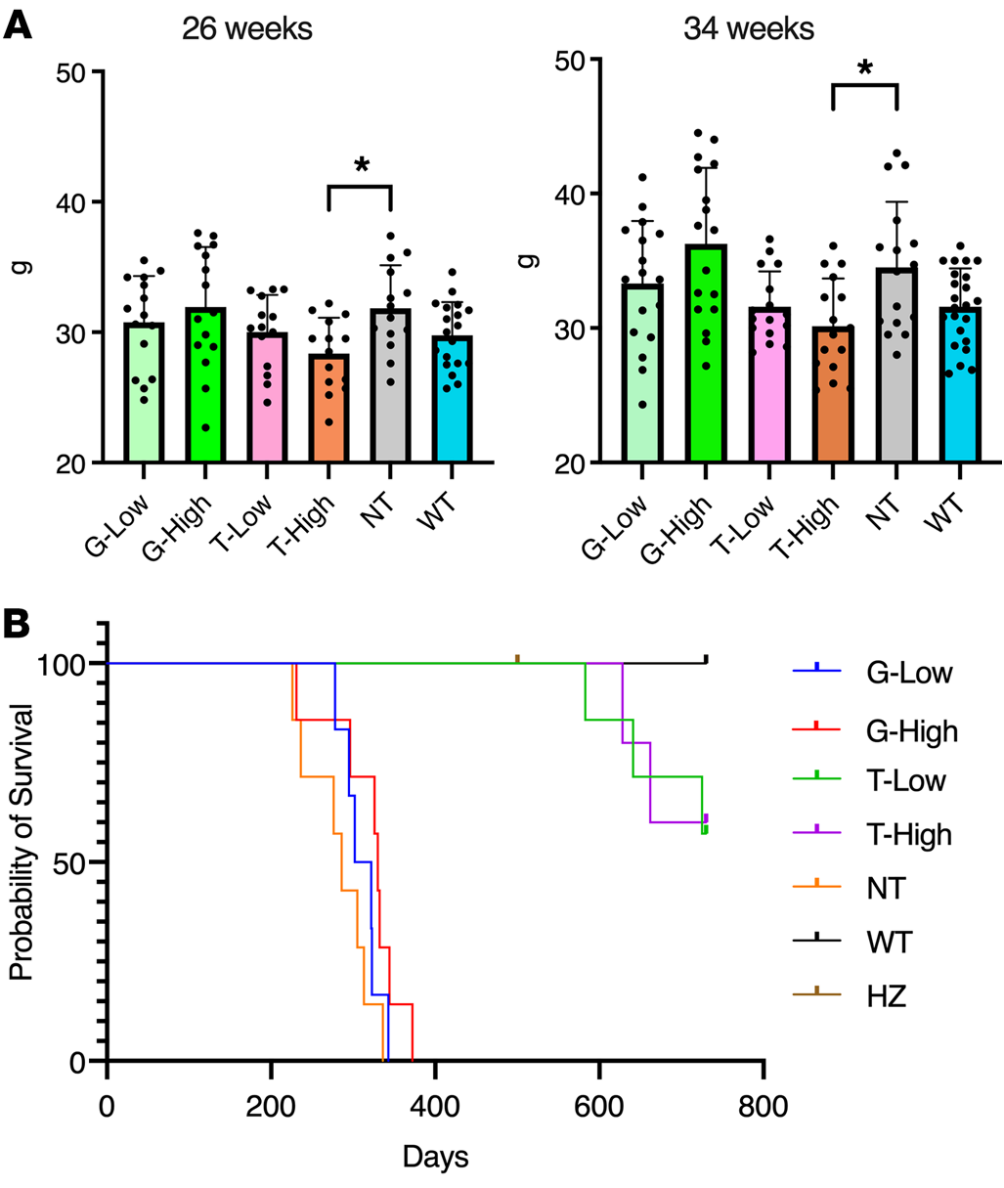
T treatment improves survival of GM1 mice. Body weight (**A**) and Kaplan-Meier survival analysis (**B**) of T-treated GM1 mice are shown. (**A**) Body weights are shown as means ± SD (26 weeks: *n* = 14 for NT and T groups, *n* = 15 for G groups, *n* = 19 for WT group; 34 weeks: *n* = 16 for NT and T groups, *n* = 17 for G groups, *n* = 23 for WT group). (**B**) For survival analysis, mice were allowed to survive until 730 days or greater than 15% body weight loss relative to the highest weight achieved for each animal (*n* = 5 for T-High group, *n* = 6 for G-Low group, *n* = 7 for G-High, T-Low, and NT groups, *n* = 9 for WT group). G-Low, 1 × 10^12^ vg/kg of AAV-β-gal treatment; G-High, 5 × 10^12^ vg/kg of AAV-β-gal treatment; T-Low, 1 × 10^12^ vg/kg of AAV-Tβ-gal treatment; T-High, 5 × 10^12^ vg/kg of AAV-Tβ-gal treatment; NT, nontreated GM1 mice; WT, wild-type mice; HZ, heterozygote mice. Significance was evaluated by a 1-way ANOVA followed by Dunnett’s multiple-comparison test and log-rank (Mantel-Cox) test. *P < 0.05 vs. NT.

The body weight at 26 and 34 weeks after treatment was higher in the NT and G groups compared with the WT and T groups (Figure 5A). The median survival of the NT group was 286 days, whereas all the mice in the WT and HZ groups survived until 730 days, which was set as the endpoint for this study (Figure 5B). The G groups showed results similar to those of the NT group, i.e., the median survival of the G-Low and G-High groups was 312 and 330 days, respectively. In contrast, all the mice in the T groups survived over 580 days. These results showed that T-mediated gene therapy extended the lifespan of the GM1 mice.

## Discussion

We demonstrated that Tβ-gal fusion enzyme expressed in the liver with an AAV vector was secreted into the blood and efficiently penetrated the brain, resulting in the complete normalization of the accumulated GM1 in the CNS, reduction of neuroinflammatory responses, improvement of neurological function, and extension of lifespan in GM1 model mice.

The accumulation of GM1 in the CNS was completely normalized, and the lifespan of the GM1 model mice was extended after T treatment. However, complete recovery of neurological function was not achieved. Because its normalization in the CNS was observed at least 1 month after T treatment (data not shown), the decrease in GM1 is thought to occur relatively soon after treatment. If irreversible neurodegeneration had already begun at the start of the treatment, it would be too late to recover all functions completely. Considering the importance of early treatment for GM1 gangliosidosis, we studied an additional group that received treatment 1 month earlier (Supplemental Figure 4). We performed a similar study in 5-week-old GM1 mice at high dose of T (T-High) and observed increased enzyme activity in blood and brain, and normalization of substrate accumulating in the brain (Supplemental Figure 4, A–C). In addition, the latency to fall in the rotarod test, the moving speed and rearing number in the open-field test, and the stride length/body length ratio in the gait assay showed improvement in the T-High group (Supplemental Figure 4, D–G). However, even with early treatment, no further improvement was observed. Thus, the reason for the lack of full recovery remains unknown, and further research is needed.

ERT, which is approved for various LSDs, is currently not available for GM1 gangliosidosis. There are two main reasons, the major one being the inability of intravenously administered enzymes to penetrate the BBB into the brain, which is required to treat GM1 gangliosidosis with predominantly neurological pathology. This is also a critical problem in other neuropathic LSDs. Recently, several ERT approaches have been developed that have therapeutic effects on the brain. One is the intracerebroventricular administration of the enzyme. This type of ERT for MPS II and neuronal ceroid lipofuscinoses type 2 has already been approved and has achieved some therapeutic effects. Another approach is the application of the BBB-penetrating system. In MPS II, a recombinant fusion protein consisting of a humanized anti-hTfR antibody and human iduronate-2-sulfatase has been approved in Japan (9). In GM1 gangliosidosis, the systemic administration of a recombinant murine β-gal fused to the plant lectin subunit B of ricin showed β-gal activity in several brain regions in model mice (45). However, only a partial reduction in GM1 levels in the brain was observed. Przybilla et al. reported a Tβ-gal–mediated ERT in GM1 model mice (46). They showed partial improvement in a behavioral test but no reduction in accumulated GM1 in the CNS.

The other issue is the stability of the human β-gal protein. Exogenous expression of the mouse β-gal protein is stable in both human and mouse cells (52), but the stability of the human β-gal protein is dependent on the protein cathepsin A (53). The functional activity of the human β-gal protein within the lysosome depends on its association with protective protein/cathepsin A in the lysosomal multienzyme complex (54). Therefore, it seems to be difficult to obtain sufficient therapeutic effect using ERT with β-gal protein alone. To overcome the limitation of ERT, we applied the BBB-penetrating system to gene therapy.

In this study, we selected the AAV vector for long-term stable expression in the liver. While patients who have anti-AAV neutralizing antibodies (NAbs) are not selected for AAV-mediated gene therapy, the proportion of the NAb-positive population increases with age (55). This is consistent with the idea that young patients should be the target of this treatment. Importantly, systemic administration of the AAV vector is already approved for gene therapy for hemophilia A and B and spinal muscular atrophy. Moreover, liver-directed gene therapy using AAV might induce systemic immunological tolerance to transgene products (56–59). The enzymatic activity of β-gal in the serum was maintained throughout the experiment, i.e., no inhibition of the treatment using antibodies against AAV and the transgene was observed. Although we have never investigated promoters other than this liver-specific promoter, these results suggested that immune tolerance might contribute to the effective treatment observed in this study.

Genetic diseases such as hemophilia and Pompe disease can cause unfavorable immune responses during treatments with ERT because of the lack of enzymatic expression in the patient (60–62). Cross-reactive immunological material–negative (CRIM-negative) patients make no enzyme protein and develop sustained high antibody titers to ERT that render the treatment ineffective. Patients with the most severe type of GM1 also make no β-gal protein, because they have a large deletion in the *GLB1* gene, and it is highly likely that they are CRIM negative. Therefore, it would be advantageous to express it in the liver to avoid immune exclusion.

In a recent study on gene therapy using AAV8 for X chromosome–linked myotubular myopathy (NCT03199469), 3 patients in the high-dose group (3 × 10^^14^^ vg/kg) died from progressive hepatic failure after the treatment, and the trial was halted. Furthermore, the patients with spinal muscular atrophy developed acute liver failure and died after infusion with onasemnogene abeparvovec (63). Although AAV itself is considered nonpathogenic, high systemic doses are hepatotoxic, making dose reduction an imperative in recent gene therapy using AAV. The maximum dose in this study was set at 5 × 10^^12^^ vg/kg, which is in the safety range and less than one-tenth the dose of commercially available AAV gene therapy drugs and was found to be sufficient for the GM1 model mice.

Differences in cell surface receptors among AAV serotypes determine their tropism. The AAV9 capsid used in this study has the ability to infect systemically, including the CNS, but transgene expression does not occur in organs other than the liver because of a liver-specific promoter. Leakage expression in the brain or the penetration of the enzyme from the blood may have occurred in the CNS, where β-gal enzyme activity was detected after G-High administration. Jeyakumar et al. reported that the permeability of the BBB is higher when GM1 mice get old (64). Nevertheless, we think that this would not affect the original purpose of this study, i.e., to compare treatment efficacy with and without anti-mTfRAb in GM1 model mice.

The binding site of anti-mTfRAb to the transferrin receptor is different from the site where endogenous transferrin binds, and thus the anti-mTfRAb was not expected to interfere with the intracellular uptake of iron. However, side effects such as mild anemia and extramedullary hematopoiesis were observed in the T-High group. However, long-term (56 weeks) follow-up results showed no further decline in HGB levels (data not shown). Therefore, we conclude that although it is necessary to monitor HGB levels, this therapy would be still valuable. Changing the binding affinity of anti-mTfRAb can also be considered to create an alternative anti-mTfRAb that does not cause anemia even at high concentrations while maintaining its efficacy.

We showed that an anti-mTfRAb–fused human β-gal excreted from AAV-transduced hepatocytes normalized the GM1 accumulation in the CNS of the model mice. The continuous secretion of the enzyme from transduced cells might have more advantages over the intermittent administration of the enzyme. This could be a revolutionary method that could be applied not only to other LSDs but also to any single-gene-caused disease that presents neurological symptoms.

## Methods

### Sex as a biological variable.

Our study examined male mice because male animals exhibited higher efficiency in AAV infection. We conducted a preliminary study to compare the differences between males and females. We found that male mice were more efficiently infected with AAV than female mice, but the treatment trend remained the same.

### Animals.

GM1 gangliosidosis model mice (GM1 mice; *β-gal^–/–^*) — a C57BL/6-based congenic strain KO mouse with β-gal deficiency (65) — and heterozygous mice (*β-gal^+/–^*) were obtained originally from the Laboratory Animal Resource Bank at the National Institutes of Biomedical Innovation, Health and Nutrition (NIBIOHN). Mice for the experiments were generated by breeding of *β-gal^+/–^* females with *β-gal^+/–^* males. Genotypes were determined using polymerase chain reaction (65).

### Animal procedures.

GM1 mice at 5 or 10 weeks of age were injected with 200 μL of the G or the T vector at 1 × 10^^12^^ (low dose) or 5 × 10^^12^^ (high dose) vg/kg via the tail vein.

A blood sample from each mouse was collected before the AAV injection and then at 6, 14, and 23 weeks by puncturing of the superficial temporal vein of the mouse using a heparinized microcapillary tube. The collected blood was placed inside a microtube and centrifuged at 10,000*g* for 10 minutes at 4°C, and the supernatants were stored as plasma samples at –80°C until all the samples had been collected. At the termination of the study (23 weeks after AAV treatment), the mice were anesthetized by isoflurane inhalation (Pfizer) and perfused with 30 mL of PBS. The brain, liver, spleen, and heart were collected for biochemical analysis. The mice were anesthetized by isoflurane inhalation and perfused with 30 mL 4% paraformaldehyde (FUJIFILM Wako Pure Chemical) followed by the collection of the brain for histological analysis. In the survival study, mice were euthanized at the humane endpoint, which was defined by 15% weight loss relative to the highest weight achieved for each animal.

### AAV vector construction and production.

The pAAV vectors carrying AAV2 ITR and the transgene expression cassette were constructed as follows. In brief, the cytomegalovirus (CMV) promoter on pAAV-CMV (TaKaRa) was replaced by the murine hepatocyte-specific promoter composed of murine α-fetoprotein enhancer, murine minimal albumin promoter, and chimeric chicken β-actin/MVM intron (mMAP), resulting in pAAV-mMAP-MCS. The human *GLB1* gene (accession P16278) was cloned into pAAV-mMAP–multi cloning site (MCS), resulting in pAAV-mMAP-*GLB1* for AAV-β-gal (referred to as G in this study). Single-chain variable fragment (scFv) of the mTfR was fused with linker at the 5′ end of *GLB1* and cloned into the pAAV-mMAP-MCS, resulting in pAAV-mMAP-TfR-*GLB1* for AAV-TfRβ-gal (referred to as T in this study). The expression unit, mMAP-TfR-*GLB1*, is large enough to be mounted on an AAV (Supplemental Figure 5). The AAV helper plasmid was constructed by replacement of the *Cap6* gene on the pR2C6 (TaKaRa) to AAV9 Cap sequence, resulting in pR2C9. AAV9 vectors were produced through triple-plasmid transfection of HEK293T cells (ATCC) and purified by 2-step chromatography with POROS Capture Select AAVX affinity resin (Thermo Fisher Scientific) and POROS 50HQ strong anion exchange resin (Thermo Fisher Scientific). The final vectors were formulated in PBS containing 350 mM NaCl and 0.01% pluoric-68.

### β-Gal activity analysis.

Frozen tissues and serum samples were used for β-gal analysis. The frozen tissues were homogenized in 9 volumes of distilled water and centrifuged at 20,000*g* for 15 minutes at 4°C. The supernatant of the frozen tissues and serum was used for the β-gal assay. The total β-gal activity was determined using 4-methylumbelliferyl-β-d-galactopyranoside as the synthetic fluorogenic substrate, as described previously (66). Briefly, 10 μg protein in tissue lysates or 10 μL serum was mixed with the substrate, which was 0.5 mM 4-methylumbelliferyl-β-d-galactopyranoside in 0.1 M sodium acetate–acetic acid buffer (pH 4.0) with 0.1 M NaCl, and the mixture was incubated at 37°C for 20 minutes. The reaction was terminated by addition of 3.98 mL stop buffer. The total β-gal assay was performed by measurement of the release of 4-methylumbelliferyl at 365 nm excitation and 460 nm emission on an RF5300PC spectrofluorophotometer (Shimadzu). Protein concentrations were determined using the Pierce BCA Protein Assay Kit (Thermo Fisher Scientific) following the manufacturer’s instructions. Enzymatic activity was expressed as either nanomoles per hour per milliliter (serum) or nanomoles per hour per milligram (tissues, normalized to total protein concentration).

### Analysis of GM1 ganglioside content.

LC-MS/MS was used for the quantification of GM1 ganglioside in the CNS. Total lipids were extracted using the Folch method (67). Briefly, tissues were homogenized with distilled water, followed by extraction with chloroform/methanol (2:1, vol/vol). The extracted samples were analyzed using LC-MS/MS (LCMS-8040, Shimadzu) as described previously (40).

### Histological analysis.

Histological analysis was performed as described previously (68). Briefly, for cryoprotection, the fixed brain was placed in 30% sucrose in PBS for 1 day at 4°C. The brain tissue was mounted onto the stage of a freezing microtome with optimal cutting temperature (OCT) compound, and the stage angle was adjusted to keep the sagittal plane of the brain horizontal. The brain tissue was cut into 20 μm–thick parasagittal sections, and stored in 0.02% sodium azide in 0.1 M phosphate buffer.

For immunohistochemistry, the sections were washed with PBS for 10 minutes twice followed by PBS-X (PBS with 0.3% Triton X-100) (68) for 30 minutes at 20°C–25°C. Primary antibodies included Alexa Fluor 488–conjugated CTX-B (1:300; Invitrogen, C34775), chicken anti-GFAP (1:500; Millipore, AB5541), rabbit anti-Iba1 (1:500; Wako, 019-19741), goat anti-LAMP1 (1:500; R&D Systems, AF4320), and mouse anti-NeuN (1:500; Millipore, MAB377), and DAPI (1:1,000; Wako, D523) was used. The sections were incubated for 7 days with a mixture of primary antibodies in PBS-XCD (PBS-X with 0.12% λ-carrageenan and 1% normal donkey serum) (68). The sections were washed with PBS-X for 10 minutes twice, then incubated for 7 days in a mixture of secondary antibodies in PBS-XCD. The following secondary antibodies and dilutions were used (all from Biotium): Alexa Fluor 555 donkey anti-mouse (1:500; 20037), Alexa Fluor 568 anti-rabbit (1:500; 20102), anti-chicken (1:500; 20104), Alexa Fluor 647 donkey anti-mouse (1:500; 20046), and anti-goat (1:500; 20048). The sections were then washed with PBS-X for 20 minutes twice and then with PBS for 20 minutes. Finally, the sections were mounted onto APS-coated glass slides, and a coverslip was applied using a mounting medium for fluorescence microscopy.

### Measurement of cytokine/chemokine levels.

MIP-1a (CCL3) and IP-10 levels were measured in the cortex using Legendplex (BioLegend) according to the manufacturer’s instructions.

### Behavioral experiments.

Mice were group-housed (5 mice per cage) under a 12-hour light/12-hour dark cycle and provided with water and food ad libitum. Rotarod testing was conducted using a rotarod apparatus (LE8205, Panlab Harvard Apparatus) as described previously (40). Briefly, animals were placed on a rotarod accelerating from 4 to 40 rpm over 5 minutes, and the latency to fall was recorded. Practice trials of 1 minute at 4 rpm were conducted for 2 days before the testing day. Testing was conducted with 1 practice trial of 1 minute at 4 rpm before the session, followed by 3 trials with 20 minutes of rest. The latency to fall for each mouse in a testing session was recorded, and the longest time on the rotarod in any of the 3 trials was reported.

Open-field experiments were conducted on a square apparatus with gray walls (O’Hara & Co. Ltd.) as described previously (40). Briefly, the total traveling distance (m), time spent in the center region (percent), moving speed (cm/s), and rearing number were recorded during the 10-minute test session.

Gait analysis was conducted on the transparent acrylic plate platform (5 cm wide, 30 cm length) under 200–250 lux brightness. Each animal was walked for at least 3 consecutive trials for image analysis. Mouse gait and entire body images were captured from under the acrylic plate using a web camera (HD Webcam C525, Logicool) and the corresponding software (version 2.51, Logicool). Body length and stride length were defined as previously reported (69, 70) and manually measured using ImageJ Fiji (version 1.0). Three trials were analyzed for each individual mouse, and 3 steps were analyzed per trial.

### Statistics.

GraphPad Prism (version 9.5.1, Dotmatics) was used for 1-way or 2-way ANOVA, Tukey’s multiple-comparison test, Dunnett’s multiple-comparison test, and Kaplan-Meier method for analysis. Significance was set at *P* less than 0.05%.

### Study approval.

The Institutional Animal Care and Use Committee of Jikei University approved all the procedures, including animal experiments and husbandry (2019-027C5).

### Data availability.

All data are available in the main text or the supplemental materials. Values for all data points in graphs are reported in the Supporting Data Values file.

## Author contributions

SKM designed and performed the majority of the experiments and analyses, and wrote the manuscript. YS performed LC-MS/MS analysis. MK, HT, and Shunsuke Iizuka designed and generated vectors. SO and HH performed histological analyses. Sayoko Iizuka performed earlier treatment group analysis. TH, TN, and AMW supervised behavioral experiments. HS and HK directed the study. TO directed the study and wrote the manuscript.

## Supplementary Material

Supplemental data

Supporting data values

## Version 1 04/08/2025

In-Press Preview

## Version 2 06/16/2025

Electronic publication
