# Supplementary material for: Adeno-associated virus expressing a blood-brain barrier–penetrating enzyme improves GM1 gangliosidosis in a preclinical model
Source: J Clin Invest. 2025 Apr 8;135(12):e180724. doi: 10.1172/JCI180724 (PMC12165788; doi:10.1172/JCI180724)
Supplement: Supplemental data [file jci-135-180724-s202.pdf]

Supplemental figure 1. β-gal enzymatic activity in serum of GM1 mice treated with T.

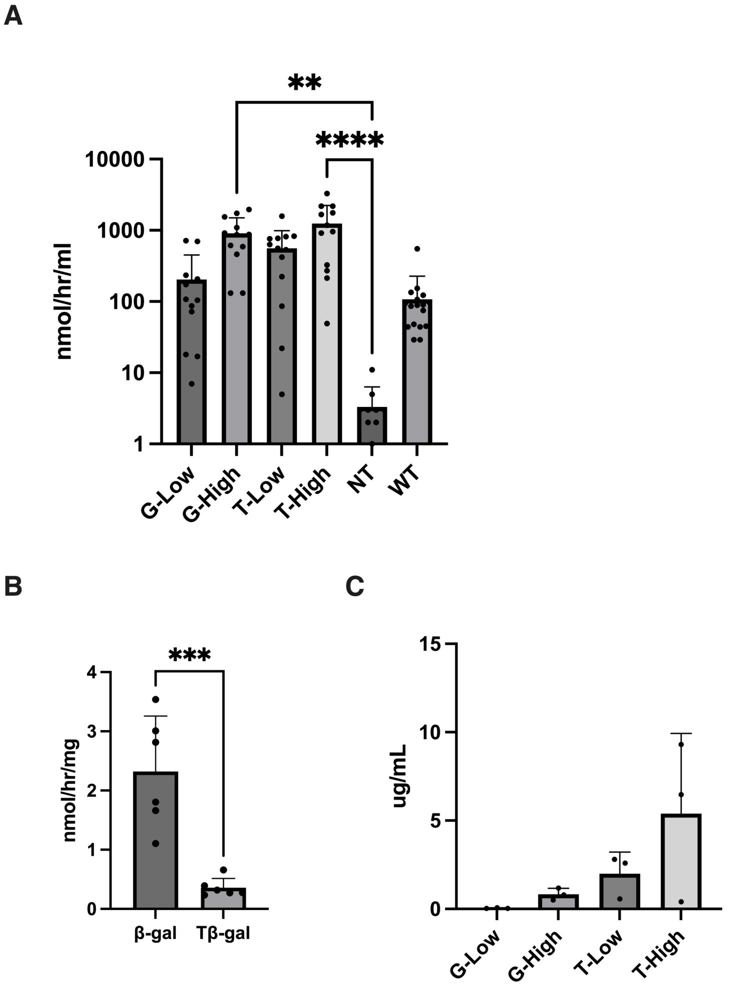

A β-gal enzyme activity at 23 weeks after treatment (n = 10 for NT group, n = 12 for G groups, n = 13 for T groups, n = 17 for WT group). **B** The specific activity of β-gal and Tβ-gal in the serum at 23 weeks after treatment (n = 6/group). Each specific activity was calculated by protein concentration and β-gal activity in the serum. **C** The β-gal protein concentration in the serum at 23 weeks after treatment (n = 3/group). The protein concentration in the serum was evaluated by ELISA using anti-β-gal antibody. In brief, a biotin-labeled anti-hGLB1 monoclonal antibody (in house; provided by JCR Pharmaceuticals) was applied to a pre-blocked streptavidin-coated plate and incubated at room temperature for one hour. After solid phase incubation, serum samples, anti-hGLB1 polyclonal antibody, and the SULFO-TAG-labeled anti rabbit antibody (Meso Scale Diagnostics) were sequentially incubated at room temperature for one hour. The intensity of electrochemiluminescence was quantified by Sector Imager 6000 (Meso Scale Diagnostics). These results are shown as means ± SDs. G-Low; 1E+12 vg/kg of AAV-β-gal treatment, G-High; 5E+12 vg/kg of AAV-β-gal treatment, T-Low; 1E+12 vg/kg of AAV-T-βgal treatment and T-High; 5E+12 vg/kg of AAV-Tβ-gal treatment, NT; non-treated-GM1 mice, WT; wild-type mice. Significance was evaluated by a one-way ANOVA followed by Dunnett's multiple comparisons test versus NT (**A**) or unpaired t test (**B**). **$p < 0.005$, ***$p < 0.001$, ****$p < 0.0001$.

Supplemental figure 2. The percentage of time spent in center at open field test.

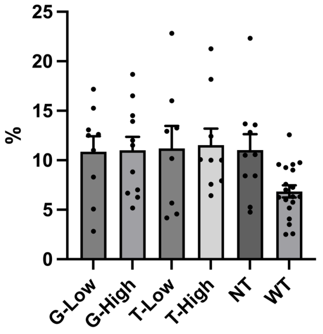

Behavioral evaluation with open field test was conducted at 23 weeks after treatment (n = 8 for T-Low group, n = 9 for G-Low and T-High groups, n = 10 for NT group, n = 11 for G-High group, n = 19 for WT group). The percentage of time spent in center region was analyzed. G-Low; 1E+12 vg/kg of AAV-β-gal treatment, G-High; 5E+12 vg/kg of AAV-β-gal treatment, T-Low; 1E+12 vg/kg of AAV-Tβ-gal treatment and T-High; 5E+12 vg/kg of AAV-Tβ-gal treatment, NT; non-treated-GM1 mice, WT; wild-type mice. The result is shown as means ± SEMs.

Supplemental figure 3. Effects of T treatment on hematopoiesis.

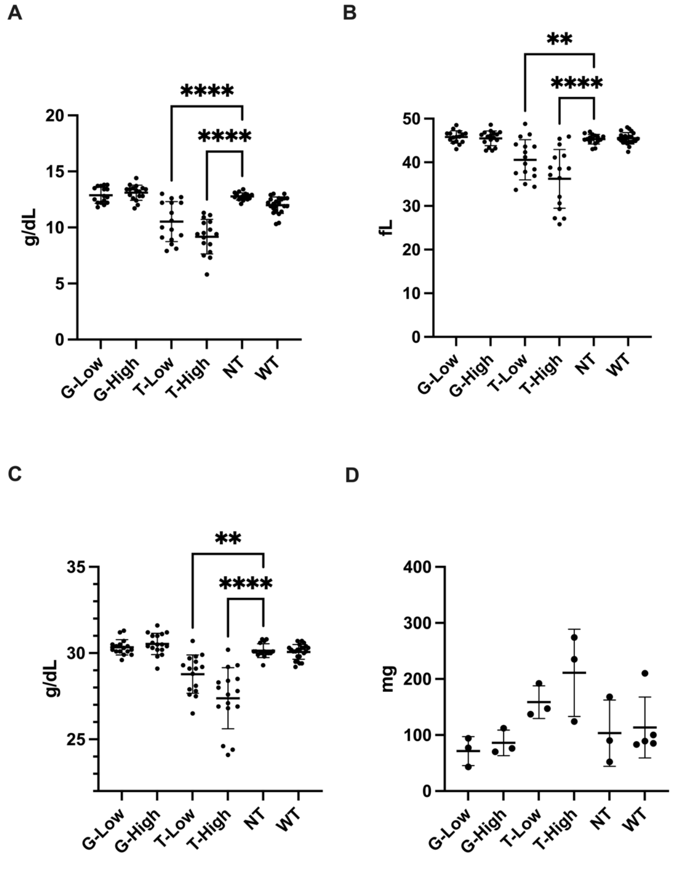

To figure out the effect of T treatment on hematopoiesis, blood counts were performed at 23 weeks after treatment. **A** HGB; Hemoglobin, **B** MCV; Mean corpuscular volume, **C** MCHC; Mean corpuscular hemoglobin, **D** Spleen weights are shown (**A-C**; n = 23 for WT, n = 16 for other groups, **D**; n = 5 for WT, n = 3 for other groups). G-Low; 1E+12 vg/kg of AAV-β-gal treatment, G-High; 5E+12 vg/kg of AAV-β-gal treatment, T-Low; 1E+12 vg/kg of AAV-T-βgal treatment and T-High; 5E+12 vg/kg of AAV-T-βgal treatment, NT; non-treated-GM1 mice, WT; wild-type mice. The results are shown as means ± SDs. Significance was evaluated by a one-way ANOVA followed by Dunnett's multiple comparisons test. **$p < 0.005$, ****$p < 0.0001$ versus NT.

Supplemental figure 4. Effects of early treatment on motor performances.

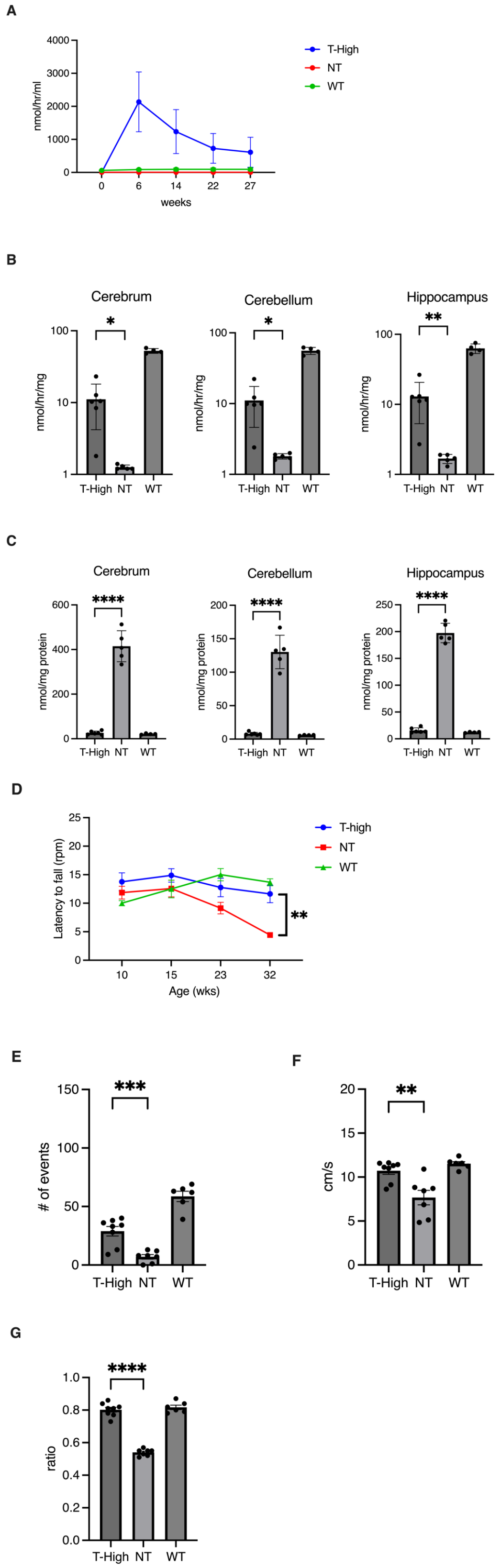

To evaluate the effectiveness of early treatment on the locomotor performance in GM1 mice, several behavioral experiments were conducted using mice treated at 5-6 weeks of age. β-gal activities in serum (**A**) (n = 8 for T-High group, n = 7 for NT group, n = 6 for WT group) and brain (**B**) (n = 6 for T-High group, n = 5 for NT group, n = 4 for WT group) were analyzed. **C** GM1 storage of the cerebrum, cerebellum, and hippocampus was evaluated by LC-MS/MS (n = 6 for T-High group, n = 5 for NT group, n = 4 for WT group). **D** Rotarod testing: highest latency achieved on 4-40 rpm accelerating rotarod over 300 s over 3 trials (n = 8 for T-High group, n = 7 for NT group, n = 6 for WT group). **E, F** Open field test. rearing number (**E**), and moving speed (**F**) were analyzed (n = 8 for T-High group, n = 7 for NT group, n = 6 for WT group). **G** Footprints were evaluated for stride length/body length (n = 8 for T-High group, n = 7 for NT group, n = 6 for WT group). T-High; 5E+12 vg/kg of AAV-T-βgal treatment, NT; non-treated-GM1 mice, WT; wild-type mice. The results are shown as means ± standard error of the means (SEM). Significance was evaluated by unpaired *t* test or two-way ANOVA followed by Sidak's multiple comparisons test. *$p < 0.05$, **$p < 0.005$, ***$p < 0.001$, and ****$p < 0.0001$.

Supplemental figure 5. Structure of pAAV-mMAP-TfR-GLB1.

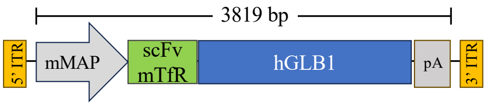

pAAV-mMAP-TfR-GLB1

The structure of the plasmid "pAAV-mMAP-TfR-GLB1" for AAV-TfRβ-gal (T) construction. ITR; AAV2 ITR, mMAP; the murine hepatocyte-specific promoter composed of murine alpha-fetoprotein enhancer, murine minimal albumin promoter, and chimeric chicken beta-actin/MVM intron, scFv mTfR and hGLB1; a single chain variable-fragment (scFv) of the mTfR was fused with human GLB1 gene. pA; bovine growth hormone polyadenylation signal.
